# Climate and seed mass drive intraspecific variation in seed longevity in storage

**DOI:** 10.1002/ajb2.70202

**Published:** 2026-05-07

**Authors:** Lea Klepka, Sascha Liepelt, Anna Bucharova

**Affiliations:** ^1^ Conservation Biology, Department of Biology Philipps University Marburg Karl‐von‐Frisch‐Straße 8 Marburg 35043 Germany

**Keywords:** artificial seed aging, ex situ conservation, intraspecific trait variation, probit analysis, seed bank, seed storage, species comparison, within‐species variability, σ (sigma)

## Abstract

**Premise:**

Conservation seed banks are essential for ex situ plant conservation, but stored seeds slowly deteriorate and lose viability. Seed longevity in storage is determined by the initial seed viability and the rate of seed viability loss. The rate of seed viability loss in storage varies between species and possibly between populations or even genotypes within species. However, the extent of this intraspecific variability and its drivers remain unclear.

**Methods:**

We investigated both inter‐ and intraspecific variability in seed longevity and its predictors in 41 common grassland species and 188 seed lots from across Europe. We exposed the seeds to simulated aging conditions (60% RH, 45°C) and used probit analysis to obtain the rate of seed viability loss (*σ*) as a measure of seed longevity. We then related *σ* to seed‐lot‐ and species‐specific factors.

**Results:**

Seed longevity (*σ*) varied significantly among seed lots within 58% of the species, and the probability of detecting such intraspecific differences increased with the number of seed lots available for a given species. This suggests that within‐species variation in seed longevity is widespread. Seed‐lot‐specific predictors explained only 14.4% of the within‐species variability. Specifically, seed longevity increased with the mean annual temperature at the seed lot origin and decreased with the seed‐lot‐specific seed mass. Across species, seed longevity differed among plant families but was unrelated to seed mass, protein, or oil content.

**Conclusions:**

Our findings highlight substantial within‐species variation in seed longevity in storage, but it is difficult to predict.

Biodiversity conservation is one of the critical global challenges, and ex situ methods play an increasingly important role in this task (Cohen et al., 1991). One of the most common ex situ methods for plant conservation is to store seeds in seed banks, dedicated facilities that store seeds of wild plant species, particularly the vulnerable ones (Peres, 2016; Wambugu et al., 2023). The stored seeds represent a snapshot of past populations and are valuable resources for species reintroductions or ecosystem restoration and for scientific research (Cochrane et al., 2007; Etterson et al., 2016; Wambugu et al., 2023). Thus, conservation seed banks aim to maintain viability for as long as possible, ideally for decades or even centuries.

Seed survival in storage depends on the seed's storage behavior and the storage conditions. Seeds are categorized by their physiological response to desiccation and low temperature. Recalcitrant seeds do not survive desiccation, making long‐term storage impossible. In contrast, orthodox seeds can survive desiccation and can be stored for months to centuries, depending on the species (Hay and Probert, 2013). Conservation seed banks aim to maintain seed viability as long as possible by storing the seeds at low temperatures and relative humidity to slow down internal chemical processes such as lipid peroxidation and DNA damage (Walters et al., 2005; Nagel et al., 2015). Seed longevity also depends on the quality and viability of the seeds (Niedzielski et al., 2009; Hay and Probert, 2013); thus, guidelines for seed banking recommend storing seed lots with high initial viability (ENSCONET, 2009; FAO, 2022; Millennium Seed Bank, 2022). Yet, even highly viable seeds stored under optimal conditions slowly deteriorate and eventually die. This mortality is faster when the seeds are stored in suboptimal conditions, for example, when large quantities of seeds that are required for large‐scale ecosystem restoration are stored in uncontrolled, ambient conditions at a farm (Merritt and Dixon, 2011). To optimize the long‐term effectiveness of seed storage—be it for conservation, research, or ecosystem restoration purposes—we need to understand which factors apart from storage conditions predict seed longevity.

Studying seed longevity presents unique challenges because it requires testing old and younger seeds from the same seed lot in the same experiment. To circumvent this obstacle, researchers have developed protocols to simulate natural aging by exposing the seeds to high temperatures and high relative humidity (Delouche and Baskin, 1973). These conditions accelerate similar chemical processes typical of natural aging during long‐term storage (Delouche and Baskin, 1973). Although this method may not perfectly replicate natural aging, the longevity of seeds in artificial aging correlates with the longevity of seeds stored in seed banks both across and within species (Probert et al., 2009; Ninoles et al., 2022). Nevertheless, artificial aging is the best method we currently have to compare seed longevity among seed lots.

Seed survival during artificial aging differs between species and, to some degree, between higher taxa such as plant families, although seed longevity often varies substantially within the same family (Walters et al., 2005; Probert et al., 2009). Studies aiming to detect general patterns in seed longevity beyond evolutionary history have yielded mixed results. Endospermic seeds are shorter‐lived in storage than non‐endospermic ones (Probert et al., 2009). The influence of seed mass is less clear; some studies suggest that seeds of small‐seeded species survive longer (Daws et al., 2006; Satyanti et al., 2018), while others report no effect of seed mass (Probert et al., 2009; Merritt et al., 2014; Davies et al., 2020). Seed longevity is also affected by climatic conditions at collection sites: Seeds from hot or dry environments generally survive longer than those from cooler or wetter conditions (Probert et al., 2009; Merritt et al., 2014; Zani and Müller, 2017). Furthermore, seed longevity should hypothetically depend on the seed's chemical composition. For example, high concentrations of certain lipids may reduce seed longevity due to ongoing lipid peroxidation in storage (Narayana Murthy and Sun, 2000; Pritchard and Dickie, 2003), yet the results of empirical studies are inconsistent (Walters et al., 2005; Probert et al., 2009; Nagel et al., 2015).

In contrast to between‐species comparison, there is less comprehensive information on within‐species variability on seed longevity in storage. Most data have come from cultivated crops or model species (Franks et al., 2018; Lee et al., 2019; Guzzon et al., 2021; Hay et al., 2021; Bizouerne et al., 2023), with only a few studies addressing natural populations of wild species (Walters et al., 2005; Kochanek et al., 2009; Mondoni et al., 2011; Merritt et al., 2014; Genna et al., 2020; White et al., 2023). These indicate that the variation in seed longevity within a species can be as substantial as the variation between species from the same ecosystem (Kochanek et al., 2009; Mondoni et al., 2011). The predictors of seed longevity on the level of populations, genotypes, or varieties appear to be similar to those driving between‐species differences: In general, lighter seeds of the same species typically survive longer than heavier seeds (Franks et al., 2019; Guzzon et al., 2018; Lee et al., 2019, but see Mira et al., 2019). Consequently, seeds of genotypes or varieties with lighter seeds tend to survive longer in storage than seed lots with heavier seeds, although this effect is inconsistent across studies (Schutte et al., 2008; Guzzon et al., 2021). The climate at the seed's origin also seems to be a critical factor, with populations from warmer and drier environments producing longer‐lived seeds than populations from colder and more humid environments (Mondoni et al., 2011, 2014; but see Kochanek et al., 2009). The differentiation in seed longevity among populations of the same species can be driven by two, nonmutually exclusive mechanisms: genetic differentiation (Mondoni et al., 2014, p. 20) or phenotypic plasticity, where parental growing conditions influence seed longevity (Sinniah et al., 1998; Kochanek et al., 2010; Mondoni et al., 2014). While the studies above provide valuable insights, we are still missing a comprehensive assessment of intraspecific variability in seed longevity across wild species, its magnitudes, and predictors.

To fill this gap, we focused on more than 40 common grassland species represented by up to 11 seed lots from across Europe. We exposed the seeds to artificial ageing, estimated their longevity, and compared the data on two levels: across seed lots within a species and across species.

We expect that (1) seed longevity under artificial ageing conditions differs between species and seed lots within species. The variation among species will be larger than the variation within species. (2) Within species, longevity will increase with mean annual temperature and annual precipitation at the seed origin and decrease with seed mass. (3) Across species, longevity will decrease with seed mass and be influenced by seed chemical composition, particularly oil content, and the plant family.

## MATERIALS AND METHODS

### Study species and seed material

We focused on 41 species common in European grasslands, belonging to 11 families distributed across the phylogenetic tree of angiosperms (Table 1). They represent a wide variety of traits, some potentially associated with seed longevity, specifically seed mass, seed oil, and protein content (Probert et al., 2009). All species have orthodox seeds; i.e., they survive desiccation and freezing during storage.

**Table 1:** Species included in the study. The number of seed lots is the number of seed lots used for the analyses (in brackets is the initial number of seed lots). 1000‐Seed mass is the mass of 1000 seeds, averaged across all seed lots of the same species. Oil and protein content were obtained from the Seed Information Database (Society for Ecological Restoration, International Network for Seed Based Restoration, and Royal Botanical Gardens Kew, 2023). Life cycle: A = annual, P = perennial, B = biennial.

Species	No. of seed lots [initial number]	1000‐Seed mass (g)	Family	Life cycle	Oil content (%)	Protein content (%)
*Achillea millefolium*	7 [10]	0.14	Asteraceae	P	27.03	33.1
*Agrostemma githago*	2 [2]	10.02	Caryophyllaceae	A	6.8	15.3
*Ajuga reptans*	0 [4]	1	Lamiaceae	P		
*Anthemis arvensis*	0 [1]	0.37	Asteraceae	A/B		
*Arrhenatherum elatius*	2 [5]	2.83	Poaceae	P	7.13	24.4
*Barbarea vulgaris*	3 [4]	0.39	Brassicaceae	A/B	31	15.6
*Bromus hordeaceus*	3 [3]	4.38	Poaceae	A		
*Bupleurum rotundifolium*	1 [1]	2.63	Apiaceae	A		
*Capsella bursa‐pastoris*	2 [2]	0.15	Brassicaceae	A	30.53	28.23
*Cardamine hirsuta*	2 [2]	0.12	Brassicaceae	A		
*Centaurea cyanus*	1 [7]	3.94	Asteraceae	A	24.67	16.57
*Centaurea jacea*	6 [8]	1.72	Asteraceae	P		
*Cerastium holosteoides*	1 [1]	0.07	Caryophyllaceae	B/P		
*Chelidonium majus*	3 [5]	0.71	Papaveraceae	B		
*Daucus carota*	8 [9]	0.83	Apiaceae	A/B	19.26	24.38
*Dianthus deltoides*	8 [8]	0.2	Caryophyllaceae	P		
*Galium wirtgenii*	0 [1]	0.63	Rubiaceae	P		
*Geranium pusillum*	0 [1]	1	Geraniaceae	A		
*Hordeum murinum*	1 [1]	15.41	Poaceae	A	1.4	23.5
*Hypochaeris radicata*	7 [7]	0.74	Asteraceae	P		
*Lamium album*	0 [2]	1.57	Lamiaceae	P		
*Lamium purpureum*	0 [3]	0.69	Lamiaceae	A		
*Lathyrus nissolia*	1 [1]	7.13	Fabaceae	A		
*Linaria vulgaris*	5 [7]	0.15	Plantaginaceae	P		
*Lotus corniculatus*	7 [7]	1.33	Fabaceae	P	6.53	36.18
*Lotus pedunculatus*	1 [2]	0.52	Fabaceae	P		
*Lychnis flos‐cuculi*	8 [8]	0.16	Caryophyllaceae	P		
*Medicago lupulina*	3 [6]	1.33	Fabaceae	A	5.45	35.57
*Papaver rhoeas*	0 [9]	0.1	Papaveraceae	A	43.47	24.53
*Plantago lanceolata*	11 [11]	1.49	Plantaginaceae	P	6.73	17.47
*Plantago media*	5 [6]	0.34	Plantaginaceae	P(/B/A)		
*Poa annua*	2 [2]	0.44	Poaceae	A/B		
*Prunella vulgaris*	9 [9]	0.77	Lamiaceae	P	21.7	20.2
*Scandix pecten‐veneris*	0 [1]		Apiaceae	A		
*Senecio vulgaris*	2 [2]	0.17	Asteraceae	A		
*Silene vulgaris*	9 [9]	0.77	Caryophyllaceae	P	2.7	18.1
*Stellaria media*	2 [3]	0.46	Caryophyllaceae	A	5.9	17.8
*Thlaspi arvense*	1 [3]	1.18	Brassicaceae	A	31.2	24.9
*Trifolium aureum*	2 [2]	0.42	Fabaceae	A		
*Trifolium campestre*	3 [5]	0.31	Fabaceae	A/B		
*Veronica chamaedrys*	7 [8]	0.14	Plantaginaceae	P		

To test for within‐species variability, most species were represented by multiple origins. The number of seed lots per species was driven by seed availability and varied strongly between species. More than half of the species were represented by at least four origins, but for six species, we obtained only one seed lot (Table 1). In total, the experiment included 188 seed lots.

The experiment required large amounts of seeds. In total, we studied 188 seed lots of 41 species, and we needed 900 seeds per seed lot. We obtained the seeds by two methods. (1) We collected at least 50 young plants in the wild and propagating them in a cold greenhouse to produce seeds (14 seed lots). (2) We bought seeds propagated on farms for restoration purposes (174 seed lots). The starting seed material for propagation on such farms is sourced from seed zones that were designated based on climatic similarity, and the farm is typically located within the same seed zone. The propagated plants thus face a similar climate as plants in natural populations in a given region (Bucharova et al., 2019). We are aware that on‐farm propagation may cause unintended selection, which could alter seed traits (Ensslin et al., 2018; Conrady et al., 2023). In the seeds we used, this effect will be rather minor because most producers are relatively new in this business, and the propagation did not take place for many generations (A. Bucharova, Philipps‐University Marburg, personal communication). Both farm‐ and greenhouse‐propagated seeds further have the advantage that the maternal plants grew under optimal conditions and the seeds were harvested when fully ripe, thus reducing variability in seed quality, a characteristic that has a massive effect on seed longevity (Sinniah et al., 1998; Sano et al., 2016; Rahman and Ellis, 2019).

### Artificial aging

All seed lots were first stored for at least 4 weeks in ambient conditions in our laboratory (approximately 21°C, 50% RH) to equilibrate seed moisture content across seed lots. For Fabaceae seeds only, we also scarified the seeds with sandpaper to allow imbibition (Baskin, 2003). To age the seeds, we used established protocols for seed longevity phenotyping (Hay et al., 2019, 2022) by exposing them to 45°C and 60% RH in a climate‐controlled cabinet (Rumed, Rubarth Apparate GmbH, Laatzen, Germany) for 0 (control group, fresh seeds), 1, 5, 9, 15, 20, 30, 40, 57, or 72 days (adjusted as described by Probert et al., 2009). These conditions are widely used to accelerate seed deterioration and enable comparisons of relative seed longevity among seed lots and species (Probert et al., 2009; Merritt et al., 2014). For each aging duration, we placed three replicates of 30 seeds each for each seed lot and aging duration into open 1.5‐mL tubes. The position of each seed lot within an aging duration was randomized. In total, we exposed each of 188 seed lots to 10 aging durations, with 3 replicates per duration (188 lots × 10 treatments × 3 replicates × 30 seeds/replicate = 169,200 seeds).

### Germination test

For germination tests after the aging treatments, we used 6‐well plates with a well diameter of 39 mm (catalogue no. 734‐2323; VWR, Darmstadt, Germany) because they use space in the climate cabinet more efficiently than single Petri dishes. For species with large seeds (*Bupleurum rotundifolium*, *Centaurea cyanus*, *Bromus hordeaceus*, *Arrhenatherum elatius*, *Lathyrus nissolia*, *Hordeum murinum*, *Hypochaeris radicata*, *Agrostemma githago*, *Poa annua*) we used 6‐cm‐diameter Petri dishes to avoid overcrowding. Petri dishes with seeds of *Bromus hordeaceus* were wrapped in aluminium foil since light slightly inhibits germination (Andersson et al., 2002).

After the intended aging duration, we transferred each replicate of seeds into one well or a Petri dish that was lined with two filter papers in a fully randomized design. We watered the seeds with a 0.025% w/v gibberellic acid solution prepared in distilled water (Fisher Chemical, Vienna, Austria) to break dormancy, sealed the plates with transparent Parafilm, and placed them in a climate‐controlled Rumed cabinet with a 14 h light at 20°C/10 h dark at 10°C.

We checked for germination every 7 days and added distilled water if necessary. When a radicle was longer than 2 mm, we considered a seed viable and removed it from the dish. For each replicate with viable seeds, we finished the trial when no more seeds germinated for at least 4 weeks. If in a replicate no seeds germinated at all for 10 weeks, we considered the seeds not viable. If samples were contaminated with a fungus, we added 1 mL of 0.0835% w/v difenoconazol solution prepared in distilled water (Duaxo Universal Pilz‐frei, COMPO GmbH Münster, Germany).

### Seed traits and climatic data

**Figure 1:**
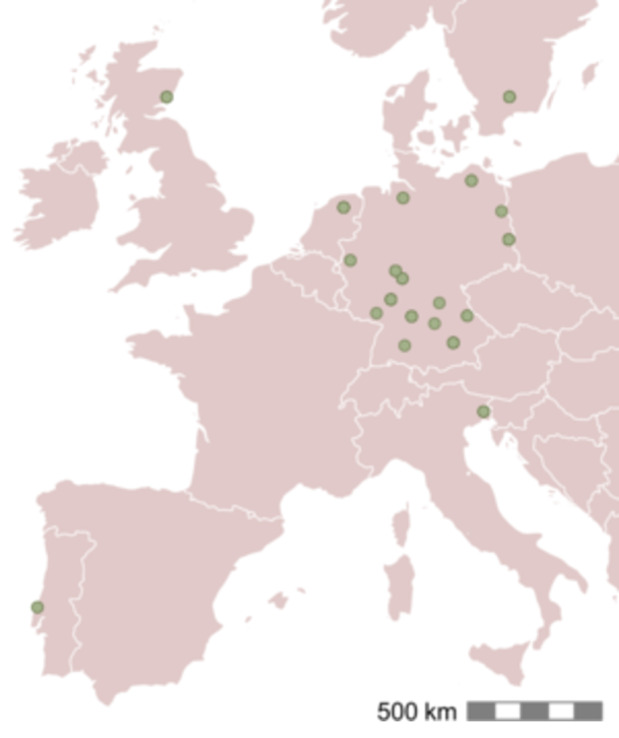
Origins of the 135 seed lots used in the study and included in the analyses. Each point represents the location of a seed producer as a proxy for the origins of the individual seed lots and thus is the origin of multiple species. In three cases, the points represent the location of our own seed collection.

For weighing each seed lot, we allowed the seed moisture content to equilibrate (ca. 21°C and 50% relative humidity, standard room conditions in our laboratory) for at least 2 weeks. We then weighed two replicates of 50 seeds each, calculated the mean, and multiplied by 20 to obtain the mass of 1000 seeds (called 1000‐mass for simplicity). We also obtained the mean annual temperature and precipitation at the source location of each seed lot from WorldClim2 using the R package raster (Fick and Hijmans, 2017). To represent population origins, we used the location of collection for seeds from wild‐collected plants. For farm‐propagated seeds, we used the location of the farm because the initial seeds used to establish the seed orchard were typically collected in the same region where the given production farm is located (Figure 1; Bucharova et al., 2019). Coordinates for the origin of each seed lot are in Table S2 (Appendix S1). Oil and protein contents of seeds for each species were obtained from the Seed Information Database (Society for Ecological Restoration, International Network for Seed Based Restoration, and Royal Botanical Gardens Kew, 2023).

### Statistical analyses

Before analyses, we calculated the initial germination percentage of each of the 188 seed lots. For some species, the initial germination percentage was higher in the short‐duration aging treatments (1 or 5 days) than in fresh seeds, likely because the short exposure broke dormancy (Mira et al., 2019; Hay et al., 2021). We thus defined “initial germination” for each seed lot as the maximum mean germination percentage at 0, 1, or 5 days in the aging treatment. We removed whole seed lots with initial germination below 50% and those particularly affected by a fungus (*N* = 39).

To analyze seed survival, we used generalized linear models (GLMs) with a quasibinomial error distribution (to account for overdispersion) and a probit link function. The probit transformation converts percentage responses to a linear scale with a normal error distribution. We modelled the proportion of germinated seeds per replicate as a function of the number of days in aging (Wolkis et al., 2025). The slope of the probit function directly describes the rate of seed viability loss, and back transformation of the slope (–1/slope) yields *σ*, the number of days needed for viability to drop by one probit (Hay et al., 2022; Klepka et al., 2025). We are aware that most comparative studies of seed longevity in storage measured seed longevity as *p*_50_, the number of days needed for viability to decrease to 50%, yet this parameter is heavily dependent on the initial seed viability (see Klepka et al., 2025 for details). While the initial viability can be affected by the environmental effects, e.g., during seed storage before the experiment, *σ* is assumed to be a genetic trait that describes a species, population, or genotype, and is thus the actual trait of interest (Hay et al., 2022). We thus decided to use *σ* as a measure of seed longevity because it is independent of the initial seed viability of given seed lots and directly describes the slope of seed viability loss (Hay et al., 2022; Klepka et al., 2025).

#### Within‐species variability

To obtain the seed longevity estimate (*σ*) for each seed lot, we related seed germination in each replicate to the aging duration in a separate probit model for each seed lot. If the model failed to give significant estimates for the slope, or if *σ* was higher than the maximum aging duration in our experiment (72 days), we excluded the seed lot from further analyses (*N* = 14), since we assumed that, in these cases, the data cover only a very short portion of the survival curve and the estimated parameters are therefore unprecise. These models yielded separate *σ*‐values for each seed lot.

We then tested whether the seed longevity (*σ*) differed between seed lots within species for the 26 species for which we had multiple seed lots that passed the initial check (initial germination >50% and significant seed‐lot‐specific *σ*). For this, we related the seed germination in each replicate to the seed lot identity, the aging duration, and their interaction as explanatory variables in a separate probit model for each species. A significant interaction between seed lot identity and aging duration indicates significantly different slopes between seed lots within species and therefore different seed longevities (*σ*). We further tested whether the probability of detecting significant differences in *σ* between seed lots within species depended on the number of seed lots representing each species in our data set. To do this, we related the presence of a significant difference between seed lots in each species (binomial yes/no) to the number of seed lots per species in a generalised linear model with binomial error distribution.

To understand drivers of within‐species variability in seed longevity in storage, we related seed‐lot‐specific longevity (*σ*) as the response variable to the seed‐lot‐specific seed mass, mean annual temperature, and precipitation at the seed lot origin as explanatory variables in a multiple linear mixed model. We included the species identity as a random factor. For this analysis, we scaled the seed mass within each species by centering and standardizing seed‐lot‐level values (mean = 0, SD = 1) to account for differences in absolute seed mass among species, and included only species that were represented by at least two seed lots.

We quantified the proportion of within‐species variance explained by the fixed effects using marginal and conditional *R* ^2^ estimates obtained with the R package MuMIn version 1.48.19 (Bartoń 2026). This allowed us to calculate the contribution of the within‐species predictors relative to the variance remaining after accounting for species identity.

#### Between‐species variability

We obtained the seed longevity estimate (*σ*) for each species, common across seed lots, regardless of whether we detected significant differences among seed lots in the previous step. For this step, we related seed germination in each replicate (and from all seed lots of the given species) to time in aging in a probit model for each species. For species with more than one seed lot, we added seed lot identity as an explanatory variable to allow for different intercepts (initial viability) in individual seed lots. These models yielded one *σ* for each species. To understand drivers of between‐species variability in seed longevity in storage, we related the log‐transformed species‐specific *σ* as the response variable to the species’ mean seed mass and plant family as explanatory variables in a multiple linear regression model. As protein and oil contents were only available for 15 study species, we evaluated their effects in a separate multiple linear regression model.

All data analyses were performed using R version 4.4.2 (2024‐10‐31 ucrt) (R Core Team 2025). We log‐transformed *σ* as the response variable to ensure that model assumptions were met. For all models, we verified that model assumptions were met by graphically evaluating the residuals (Zuur et al., 2010).

## RESULTS

**Figure 2:**
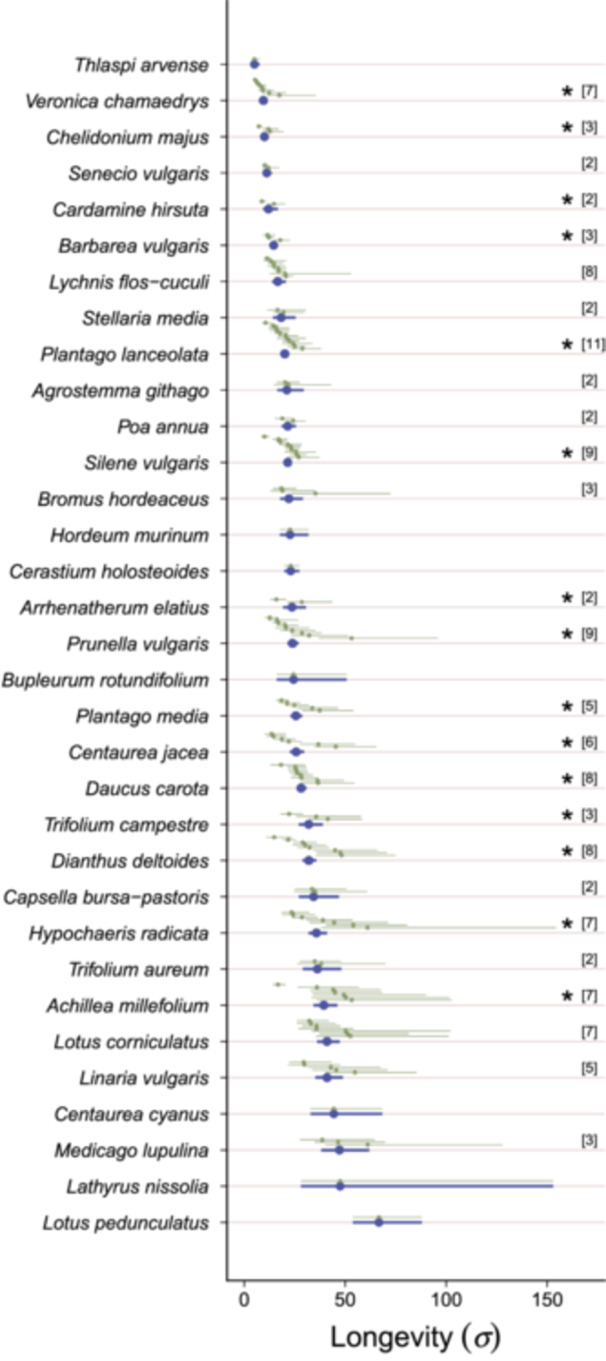
Estimated seed longevity (*σ*) and 95% confidence intervals for 33 species and 135 seed lots. Blue points and bars represent species‐level estimates; green points and bars show seed‐lot‐level estimates. Asterisks mark significant differences (*P* < 0.05) between seed lots within species. Numbers in brackets indicate the number of seed lots for species represented by multiple seed lots.

The estimated seed longevity (*σ*) significantly varied among seed lots for 15 of the 26 species (58%) for which we had multiple seed lots (Figure 2; Appendix S1: Table S1). The probability of detecting significant differences among seed lots within species increased with the number of included seed lots (*R* ^2^ = 0.20, *P* = 0.016; Appendix S1: Figure S1).

**Figure 3:**
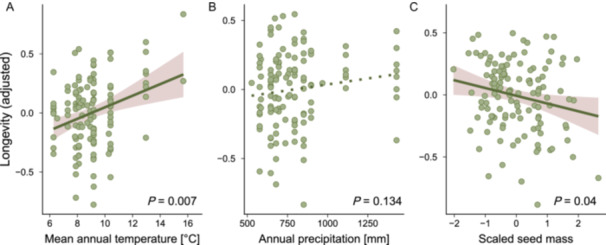
Relationship between seed longevity (*σ*) of each seed lot, seed‐lot‐specific seed traits, and environmental variables. Each point represents a seed lot (*N* = 127). Seed longevity was adjusted for species identity. (A) Mean annual temperature, (B) annual precipitation at the origins of the seed lots, and (C) seed‐lot‐specific seed mass (scaled). For full model results, see Table 2.

Within‐species variation was substantial and highest in *Prunella vulgaris*, where *σ* varied more than 4‐fold (Figure 2), specifically between 12.66 and 53.14 days. This within‐species variability was hard to predict because seed‐lot‐specific predictors explained only 14.4% of the variability among seed lots, after we corrected for species identity. Specifically, seed longevity decreased with seed mass and increased with the mean annual temperature at the seed lot origin, but was unrelated to the mean annual precipitation (Table 2, Figure 3).

**Table 2:** Intraspecific variability in seed longevity in storage, results of the linear mixed model testing the effect of seed‐lot‐specific 1000‐seed mass, mean annual temperature, and annual precipitation on seed‐lot‐specific seed longevity in storage with species identity as random factor. Analysis of variance with type III error. Significant effects (*P* < 0.05) are in bold. The marginal and conditional *R* ^2^ were 0.05 and 0.72, respectively.

Fixed effect	df	*χ* ^2^	*P*
Seed‐lot‐specific seed mass	1	4.23	**0.040**
Mean annual temperature	1	7.30	**0.007**
Annual precipitation	1	2.25	0.134

**Figure 4:**
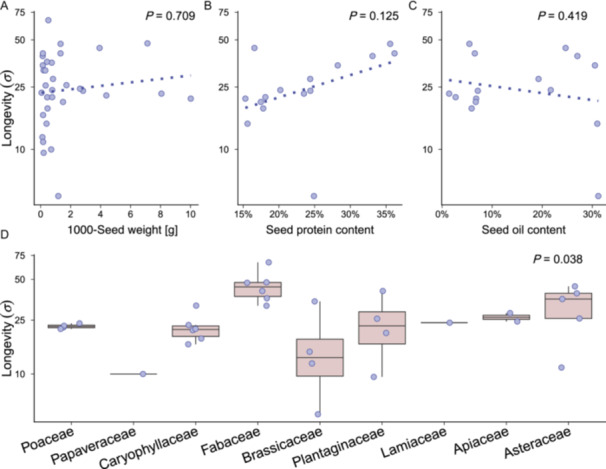
Relationship between species seed longevity (*σ*) and species‐specific seed traits. Each point represents one species. (A) Seed mass (1000‐seed mass), average across seed lots of the same species (*N* = 33); species‐specific (B) protein (*N* = 15) and (C) oil content in the seeds (*N* = 15); and (D) plant families (*N* = 33, ordered according to angiosperm phylogeny of the Open Tree of Life; OpenTreeOfLife et al., 2019). The dashed lines indicate nonsignificant relationships. For full model results, see Table 3.

The estimated seed longevity also varied among species (Figure 2), and species identity explained 66% of the total variability among seed lots. The average seed longevity was shortest for *Thlaspi arvense* (*σ* = 5.04 days) and longest for *Lotus pedunculatus* (*σ* = 66.70 days). The species seed longevity was unrelated to the species‐specific seed mass (Table 3A, Figure 4A) or the seed oil and protein content (Table 3B, Figure 4B,C), but we found significant differences between plant families, with Papaveraceae being the shortest‐lived and Fabaceae having the highest longevities (Table 3A, Figure 4D).

**Table 3:** Interspecific variability in seed longevity in storage, results of the linear model testing the effect of (A) family, species‐specific 1000‐seed mass, and (B) protein and oil content on species‐specific seed longevity. Analysis of variance with type III error. Significant effects (*P* < 0.05) are highlighted in bold.

	df	Resid.df	*F*	*P*	*R* ^2^
A		22			0.49
Family	8		2.57	**0.038**	
Species‐specific seed mass	1		0.14	0.709	
B		12			0.22
Protein content	1		0.77	0.125	
Oil content	1		0.70	0.419	

## DISCUSSION

Effective conservation of seeds in ex situ seed banks requires an understanding of seed storage behavior not only for individual species but also for seed lots for the same species. We found, for multiple species common in European grasslands, that seed lots of the same species commonly differ in seed longevity assessed under simulated aging. This intraspecific variability was partially explained by seed mass and mean annual temperature at the seed lot origin, but the predictive power of these variables was rather low (2.1% explained variability across all species). On the other hand, species identity was a strong predictor of seed longevity, explaining 66% of the variability among seed lots. Our results thus suggest that species‐level mean seed longevity can be predicted with reasonable confidence, but predicting seed‐lot‐specific seed longevity that deviates around these means remains challenging.

### Intraspecific variability in seed longevity

Seed longevity under artificial aging conditions varied considerably within‐species, with seed‐lot‐specific longevity differing significantly in 15 of 26 species with multiple seed lots. The probability of detecting significant differences among seed lots within a species increased with the number of seed lots included in the study. This pattern suggests that with more seed lots available, significant differences would likely be detected in even more species. Consequently, within‐species differences in seed longevity in storage are likely very common.

Within‐species, seed longevity under artificial aging conditions was partially explained by the mean annual temperature at the collection site, a pattern that was reported previously (Mondoni et al., 2011). However, because we assessed seed longevity under elevated temperatures to accelerate aging, this relationship may also reflect increased tolerance to higher temperatures in seeds originating from warmer climates. Nevertheless, other studies finding similar relationships attributed such a positive correlation between temperature and seed longevity mainly to the species identity because they had species represented by single seed lots (Probert et al., 2009; Merritt et al., 2014). We did not find any effect of the annual precipitation on seed longevity, which contrasts with previous studies, both within and across species (Probert et al., 2009; Merritt et al., 2014; White et al., 2023). This lack of a detectable effect is likely because the mean annual precipitation was correlated with mean annual temperature (*r* = 0.34), and because we used multivariate models, we were not able to tease apart the effect of the climate variables.

The mean annual temperature and precipitation as climatic predictors represent broad, long‐term averages and do not capture short‐term climatic extremes or variability between individual years. Yet, such extreme weather events are increasingly important under climate change (Ummenhofer and Meehl, 2017) and could potentially affect seed longevity, introducing variability that we did not account for in our study. Incorporating measures of climatic variability or extreme weather events may further improve predictions of intraspecific variability in seed longevity.

We found a negative relationship between the seed‐lot‐specific seed mass and seed longevity, with lighter seeds surviving significantly longer. Previous studies primarily tested the relationship between seed mass and longevity across species, yielding mixed results (Pritchard and Dickie, 2003; Probert et al., 2009; Merritt et al., 2014; Satyanti et al., 2018; Davies et al., 2020). Similarly, research on variability within individual species has also produced inconsistent findings (Schutte et al., 2008; Franks et al., 2018; Mira et al., 2019; Genna et al., 2020; Guzzon et al., 2018, 2021). These inconsistent study findings underscore that seed mass remains an unreliable predictor and highlight the complexity of seed longevity and its drivers. Nevertheless, our results provide new evidence suggesting that, for a variety of wild grassland species, lighter seeds may have a slight but consistent advantage over heavier seeds of the same species in long‐term storage survival.

The intraspecific variability in seed longevity may be driven by two mutually non‐exclusive mechanisms: phenotypic plasticity or genetic differentiation. For example, Mondoni et al. (2011) detected that seeds collected in populations in warmer environments survived longer in artificial aging than conspecific seeds collected in cooler environments. A large proportion of this effect was caused by phenotypic plasticity, but the pattern was still significant, although weaker, in the second generation grown in a common environment, which indicates that the effect is partially heritable and thus genetic (Mondoni et al., 2014). Indeed, genes associated with seed longevity in storage were identified in some crops and model species (Nagel et al., 2015; Renard et al., 2020; Raquid et al., 2021; Bizouerne et al., 2023). Our study worked with seeds that were produced in the region of origin after seed collection in natural sites; we thus cannot discriminate whether the observed effects are plastic or genetic. However, from a conservation‐management perspective, this distinction may be secondary. The climate at the seed source appears to be the ultimate driver of the observed differences, regardless of the underlying mechanism. The finding that seeds from warmer regions survive longer under artificial aging conditions has implications for seed bank management. Importantly, our approach mirrors the reality of conservation seed banks, where seed lots of wild plants are typically acquired from wild populations rather than from propagation under standardized conditions (FAO, 2022). While we do not claim to separate genetic from environmental causes, our findings provide practical predictors of relative differences in seed longevity that are immediately applicable to the management of conservation seed banks.

After accounting for species identity, within‐species predictors explained 14.4% of the remaining variability among seed lots. Still, species identity was the strongest predictor of seed longevity and explained 66.9%. Our findings align with those of Kochanek et al. (2009). On a continental scale, they collected data on seed longevity in seven species represented by 2–8 populations, with populations within species largely aggregated in a specific biome. Here, the species identity explained 69.1% (adjusted *R* ^2^) of the variability among seed lots, leaving 30% to variation among seed lots within species (own analysis of the data presented in Kochanek et al., 2009). In contrast, a study by Mondoni et al. (2011) found that the intraspecific variability in seed longevity was much larger than the interspecific one. On a regional scale, they examined the seed longevity of six ecologically similar species that were each represented by two populations in contrasting environments, specifically high and low altitudes. Here, species identity explained only 6% of variability, leaving an astonishing 94% variation within species (own analysis of the data presented in Mondoni et al., 2011). In our study, we detected that species explained about two‐thirds of the variability in seed longevity among seed lots, yet this number might be underestimated because we excluded the species with the most long‐lived seeds for methodological reasons (see Materials and Methods).

Comparison of our results with the findings of previous studies is complicated by the use of different measures of seed longevity. Here, we used *σ*, the rate of seed viability loss, which is independent of the initial seed viability and thus well suited for comparative studies across seed lots and species. The vast majority of previous studies used *p*_50_, the time until seed viability decreases to half (e.g., Probert et al., 2009; Mondoni et al., 2011; Satyanti et al., 2018). The metric *p*_50_ integrates the rate of viability loss (*σ*) and the initial viability (*K*_i_). As highlighted previously (Hay et al., 2019, 2022; Klepka et al., 2025), using *p*_50_ can be advantageous due to the intuitive interpretability of *p*_50_, yet it is heavily dependent on initial seed viability at the start of the experiment (Klepka et al., 2025). For example, using seeds with an initial viability ranging from 85 to 99.9%, but with the same rate of seed viability loss (*σ*), results in a 3‐fold variation in *p*_50_. We therefore used *σ* rather than *p*_50_ because *σ* is the estimate that represents inherent differences between genotypes or populations the best (Hay et al., 2019; Klepka et al., 2025). Direct comparison between our study and many previous studies is thus complex, unless raw viability data are available to calculate *σ*. To facilitate comparison, we report *K*_i_ for all seed lots in Appendix S1: Table S2.

### Interspecific variability in seed longevity

Across species, the only variable that significantly affected seed longevity was the plant family, with the observed differences between families being roughly in line with previous studies (Walters et al., 2005; Probert et al., 2009; Mondoni et al., 2011; Merritt et al., 2014): Fabaceae seeds were relatively long‐lived, a pattern consistent with previous studies (Merritt et al., 2014). Variability within Asteraceae was high (Walters et al., 2005; Merritt et al., 2014), and three of the six shortest‐lived species in our study belonged to the Brassicaceae family (*Thlaspi arvense*, *Cardamine hirsuta*, *Barbarea vulgaris*).

Neither seed mass nor the composition of chemicals evaluated in this study influenced seed longevity in our artificial aging experiment. In general, species‐specific seed mass seems to be an unreliable predictor of seed longevity under artificial aging; effects reported previously varied strongly from negative, to no effect to rarely, positive (Pritchard and Dickie, 2003; Probert et al., 2009; Merritt et al., 2014; Satyanti et al., 2018; Davies et al., 2020). Similarly, the chemical composition of the seeds did not predict seed longevity, which agrees with previous empirical studies of wild species (Walters et al., 2005; Kochanek et al., 2009). The expected effect of the chemical composition on seed longevity in storage, especially oil content, is based on theoretical expectations and comparisons among oil crops, cereals, and legumes (Narayana Murthy and Sun, 2000; Pritchard and Dickie, 2003; Nagel et al., 2015), but the empirical evidence provided by our study and others shows that it is likely not true for wild species.

## CONCLUSIONS

Our study demonstrates that for most species, the rate of seed viability loss under artificial aging conditions varies substantially. Conservation seed banks sometimes design viability monitoring of individual seed lots based on species identity and initial seed viability only, assuming that seed lots of the same species lose viability at the same rate when stored in the same conditions (Hay and Whitehouse, 2017). Our data show that this assumption is not valid and support existing concerns that this method might be suboptimal.

## AUTHOR CONTRIBUTIONS

L.K.: investigation, formal analysis, data curation, writing original draft, visualization, funding acquisition. S.L.: investigation, review and editing. A.B.: conceptualization, methodology, resources, review and editing, supervision.

## Supporting information


**Appendix S1.** Supporting tables and figures.
**Figure S1.** Relationship between the number of seed lots per species and the probability of detecting significant differences among seed lots within species. The line shows a logistic regression with the 95% confidence interval.
**Table S1.** Species‐specific seed survival models. Bold type indicates a significant effect of the parameter. A significant interaction between the seed lot identity and the aging duration means significantly different seed longevities between seed lots within species.
**Table S2.** Seed lot information for each species and seed‐lot‐specific seed survival metrics. *K*_i_ is given on the probit scale and was also back‐transformed on the percentage scale.

## Data Availability

Raw germination data and seed‐lot‐specific seed mass were deposited in Zenodo (https://zenodo.org/records/19185757).
