# Supplementary material for: Climate and seed mass drive intraspecific variation in seed longevity in storage
Source: Am J Bot. 2026 May 7;113(5):e70202. doi: 10.1002/ajb2.70202 (PMC13206544; doi:10.1002/ajb2.70202)

**Appendix S1.** Supporting tables and figures.

**Table** **S1**. Species-specific seed survival models. Bold type indicates a significant effect of the parameter. A significant interaction between the seed lot identity and the aging duration means significantly different seed longevities between seed lots within species.

| **Species** | **Number  of seed lots** | **Aging duration** | | |  | **Seed lot identity** | | |  | **Seed lot × Aging** | | |
| --- | --- | --- | --- | --- | --- | --- | --- | --- | --- | --- | --- | --- |
|  |  | **df** | ***F*** | ***P*** |  | **df** | ***F*** | ***P*** |  | **df** | ***F*** | ***P*** |
| *Achillea millefolium* | 7 | 1 | 20.05 | **<0.001** |  | 6 | 1.24 | 0.290 |  | 6 | 5.96 | **<0.001** |
| *Agrostemma githago* | 2 | 1 | 37.33 | **<0.001** |  | 1 | 0.24 | 0.623 |  | 1 | 0.07 | 0.788 |
| *Arrhenatherum elatius* | 2 | 1 | 66.81 | **<0.001** |  | 1 | 1.10 | 0.298 |  | 1 | 6.41 | **0.014** |
| *Barbarea vulgaris* | 3 | 1 | 124.86 | **<0.001** |  | 2 | 56.90 | **<0.001** |  | 2 | 5.52 | **0.006** |
| *Bromus hordeaceus* | 3 | 1 | 16.95 | **<0.001** |  | 2 | 11.07 | **<0.001** |  | 2 | 2.49 | 0.089 |
| *Capsella bursa-pastoris* | 2 | 1 | 30.59 | **<0.001** |  | 1 | 1.98 | 0.165 |  | 1 | 0.03 | 0.871 |
| *Cardamine hirsuta* | 2 | 1 | 194.27 | **<0.001** |  | 1 | 4.24 | **0.044** |  | 1 | 8.65 | **0.005** |
| *Centaurea jacea* | 6 | 1 | 50.24 | **<0.001** |  | 5 | 7.59 | **<0.001** |  | 5 | 9.85 | **<0.001** |
| *Chelidonium majus* | 3 | 1 | 101.61 | **<0.001** |  | 2 | 19.83 | **<0.001** |  | 2 | 5.23 | **0.007** |
| *Daucus carota* | 8 | 1 | 84.95 | **<0.001** |  | 7 | 11.50 | **<0.001** |  | 7 | 2.50 | **0.017** |
| *Dianthus deltoides* | 8 | 1 | 99.55 | **<0.001** |  | 7 | 5.94 | **<0.001** |  | 7 | 8.28 | **<0.001** |
| *Hypochaeris radicata* | 7 | 1 | 80.70 | **<0.001** |  | 6 | 10.98 | **<0.001** |  | 6 | 5.19 | **<0.001** |
| *Linaria vulgaris* | 5 | 1 | 23.02 | **<0.001** |  | 4 | 11.62 | **<0.001** |  | 4 | 2.09 | 0.086 |
| *Lotus corniculatus* | 7 | 1 | 26.03 | **<0.001** |  | 6 | 4.06 | **0.001** |  | 6 | 1.58 | 0.155 |
| *Lychnis flos-cuculi* | 8 | 1 | 82.17 | **<0.001** |  | 7 | 18.94 | **<0.001** |  | 7 | 1.98 | 0.059 |
| *Medicago lupulina* | 3 | 1 | 23.12 | **<0.001** |  | 2 | 9.84 | **<0.001** |  | 2 | 1.21 | 0.305 |
| *Plantago lanceolata* | 11 | 1 | 123.03 | **<0.001** |  | 10 | 12.22 | **<0.001** |  | 10 | 5.14 | **<0.001** |
| *Plantago media* | 5 | 1 | 56.65 | **<0.001** |  | 4 | 22.08 | **<0.001** |  | 4 | 5.59 | **<0.001** |
| *Poa annua* | 2 | 1 | 94.97 | **<0.001** |  | 1 | 0.02 | 0.877 |  | 1 | 2.47 | 0.122 |
| *Prunella vulgaris* | 9 | 1 | 37.96 | **<0.001** |  | 8 | 4.85 | **<0.001** |  | 8 | 6.50 | **<0.001** |
| *Senecio vulgaris* | 2 | 1 | 149.25 | **<0.001** |  | 1 | 2.91 | 0.093 |  | 1 | 0.81 | 0.372 |
| *Silene vulgaris* | 9 | 1 | 139.81 | **<0.001** |  | 8 | 12.98 | **<0.001** |  | 8 | 7.27 | **<0.001** |
| *Stellaria media* | 2 | 1 | 46.77 | **<0.001** |  | 1 | 1.25 | **0.268** |  | 1 | 0.31 | 0.579 |
| *Trifolium aureum* | 2 | 1 | 29.80 | **<0.001** |  | 1 | 5.75 | **0.020** |  | 1 | 0.12 | 0.732 |
| *Trifolium campestre* | 3 | 1 | 87.20 | **<0.001** |  | 2 | 2.68 | 0.074 |  | 2 | 4.83 | **0.010** |
| *Veronica chamaedrys* | 7 | 1 | 128.71 | **<0.001** |  | 6 | 10.05 | **<0.001** |  | 6 | 5.46 | **<0.001** |

**Table S2**. Seed lot information for each species and seed-lot-specific seed survival metrics. *K*_i_ is given on the probit scale and was also back-transformed on the percentage scale.

| **Species** | **Seed lot number** | **Seed source** | **Longitude** | **Latitude** | ***K*_i_ (±SE)** | ***K*_i_ (%)** | **Initial germination (%)** |
| --- | --- | --- | --- | --- | --- | --- | --- |
| *Achillea millefolium* | 1 | farm propagation | 8.58 | 49.84 | 1.42 ± 0.21 | 0.92 | 0.97 |
| *Achillea millefolium* | 2 | farm propagation | 6.21 | 52.96 | 1.78 ± 0.16 | 0.96 | 1.00 |
| *Achillea millefolium* | 3 | farm propagation | 11.70 | 48.37 | 1.36 ± 0.15 | 0.91 | 0.92 |
| *Achillea millefolium* | 4 | farm propagation | 9.14 | 50.55 | 1.19 ± 0.18 | 0.88 | 0.94 |
| *Achillea millefolium* | 5 | farm propagation | 14.51 | 56.71 | 1.60 ± 0.19 | 0.95 | 1.00 |
| *Achillea millefolium* | 6 | farm propagation | −2.66 | 56.70 | 1.54 ± 0.21 | 0.94 | 0.94 |
| *Achillea millefolium* | 7 | farm propagation | 14.12 | 52.83 | 1.25 ± 0.15 | 0.90 | 0.92 |
| *Agrostemma githago* | 1 | farm propagation | 8.58 | 49.84 | 1.55 ± 0.20 | 0.94 | 1.00 |
| *Agrostemma githago* | 2 | farm propagation | 6.21 | 52.96 | 1.77 ± 0.41 | 0.96 | 0.98 |
| *Arrhenatherum elatius* | 1 | farm propagation | 13.22 | 46.04 | 0.38 ± 0.15 | 0.65 | 0.74 |
| *Arrhenatherum elatius* | 2 | farm propagation | 11.70 | 48.37 | 0.18 ± 0.10 | 0.57 | 0.61 |
| *Barbarea vulgaris* | 1 | farm propagation | 8.58 | 49.84 | 0.17 ± 0.11 | 0.57 | 0.67 |
| *Barbarea vulgaris* | 2 | farm propagation | 6.21 | 52.96 | 2.33 ± 0.18 | 0.99 | 0.98 |
| *Barbarea vulgaris* | 3 | farm propagation | 11.70 | 48.37 | 1.57 ± 0.17 | 0.94 | 0.93 |
| *Bromus hordeaceus* | 1 | farm propagation | 8.58 | 49.84 | -0.25 ± 0.16 | 0.40 | 0.53 |
| *Bromus hordeaceus* | 2 | farm propagation | 11.70 | 48.37 | 0.94 ± 0.17 | 0.83 | 0.83 |
| *Bromus hordeaceus* | 3 | farm propagation | 9.61 | 49.26 | 0.61 ± 0.23 | 0.73 | 0.94 |
| *Bupleurum rotundifolium* | 1 | farm propagation | 9.61 | 49.26 | -0.32 ± 0.17 | 0.37 | 0.50 |
| *Capsella bursa-pastoris* | 1 | farm propagation | 6.21 | 52.96 | 1.38 ± 0.19 | 0.92 | 0.97 |
| *Capsella bursa-pastoris* | 2 | farm propagation | −2.66 | 56.70 | 0.99 ± 0.21 | 0.84 | 0.83 |
| *Cardamine hirsuta* | 1 | greenhouse | 6.54 | 51.16 | 1.94 ± 0.20 | 0.97 | 0.98 |
| *Cardamine hirsuta* | 2 | greenhouse | 8.81 | 50.81 | 1.35 ± 0.20 | 0.91 | 0.94 |
| *Centaurea cyanus* | 1 | farm propagation | 8.58 | 49.84 | 0.04 ± 0.11 | 0.51 | 0.57 |
| *Centaurea jacea* | 1 | farm propagation | 8.58 | 49.84 | 0.07 ± 0.12 | 0.53 | 0.59 |
| *Centaurea jacea* | 2 | farm propagation | 6.21 | 52.96 | -0.04 ± 0.12 | 0.49 | 0.67 |
| *Centaurea jacea* | 3 | farm propagation | 13.22 | 46.04 | 0.01 ± 0.10 | 0.50 | 0.53 |
| *Centaurea jacea* | 4 | farm propagation | 11.70 | 48.37 | 0.09 ± 0.14 | 0.54 | 0.66 |
| *Centaurea jacea* | 5 | farm propagation | 14.48 | 51.87 | 0.68 ± 0.14 | 0.75 | 0.86 |
| *Centaurea jacea* | 6 | farm propagation | 14.51 | 56.71 | 0.66 ± 0.11 | 0.74 | 0.78 |
| *Cerastium holosteoides* | 1 | greenhouse | 8.81 | 50.81 | 1.52 ± 0.12 | 0.94 | 0.98 |
| *Chelidonium majus* | 1 | farm propagation | 8.58 | 49.84 | 0.07 ± 0.15 | 0.53 | 0.51 |
| *Chelidonium majus* | 2 | farm propagation | 6.21 | 52.96 | 1.26 ± 0.15 | 0.90 | 0.92 |
| *Chelidonium majus* | 3 | farm propagation | 9.61 | 49.26 | 0.13 ± 0.13 | 0.55 | 0.56 |
| *Daucus carota* | 1 | farm propagation | 8.58 | 49.84 | 0.04 ± 0.07 | 0.52 | 0.53 |
| *Daucus carota* | 2 | farm propagation | 6.21 | 52.96 | -0.10 ± 0.12 | 0.46 | 0.54 |
| *Daucus carota* | 3 | farm propagation | 13.22 | 46.04 | 0.44 ± 0.11 | 0.67 | 0.60 |
| *Daucus carota* | 4 | farm propagation | 11.70 | 48.37 | 1.03 ± 0.10 | 0.85 | 0.83 |
| *Daucus carota* | 5 | farm propagation | 14.48 | 51.87 | 0.51 ± 0.08 | 0.70 | 0.76 |
| *Daucus carota* | 6 | farm propagation | 9.61 | 49.26 | 0.43 ± 0.08 | 0.67 | 0.70 |
| *Daucus carota* | 7 | farm propagation | 14.51 | 56.71 | 0.59 ± 0.10 | 0.72 | 0.68 |
| *Daucus carota* | 8 | farm propagation | −2.66 | 56.70 | 0.12 ± 0.16 | 0.55 | 0.74 |
| *Dianthus deltoides* | 1 | farm propagation | 8.58 | 49.84 | 0.74 ± 0.08 | 0.77 | 0.84 |
| *Dianthus deltoides* | 2 | farm propagation | 12.61 | 53.87 | 1.53 ± 0.17 | 0.94 | 0.96 |
| *Dianthus deltoides* | 3 | farm propagation | 6.21 | 52.96 | 1.47 ± 0.15 | 0.93 | 0.99 |
| *Dianthus deltoides* | 4 | farm propagation | 11.70 | 48.37 | 1.58 ± 0.16 | 0.94 | 1.00 |
| *Dianthus deltoides* | 5 | farm propagation | 14.48 | 51.87 | 0.73 ± 0.09 | 0.77 | 0.80 |
| *Dianthus deltoides* | 6 | farm propagation | 9.14 | 50.55 | 1.18 ± 0.12 | 0.88 | 0.92 |
| *Dianthus deltoides* | 7 | farm propagation | −2.66 | 56.70 | 1.47 ± 0.28 | 0.93 | 0.98 |
| *Dianthus deltoides* | 8 | farm propagation | 12.40 | 49.28 | 1.32 ± 0.14 | 0.91 | 0.98 |
| *Hordeum murinum* | 1 | greenhouse | 8.81 | 50.81 | 0.85 ± 0.16 | 0.80 | 0.89 |
| *Hypochaeris radicata* | 1 | farm propagation | 6.21 | 52.96 | 1.38 ± 0.20 | 0.92 | 0.96 |
| *Hypochaeris radicata* | 2 | farm propagation | 13.22 | 46.04 | 1.58 ± 0.21 | 0.94 | 0.97 |
| *Hypochaeris radicata* | 3 | farm propagation | 11.70 | 48.37 | 0.30 ± 0.10 | 0.62 | 0.59 |
| *Hypochaeris radicata* | 4 | farm propagation | 14.48 | 51.87 | 1.06 ± 0.12 | 0.86 | 0.93 |
| *Hypochaeris radicata* | 5 | farm propagation | 9.61 | 49.26 | 0.87 ± 0.14 | 0.81 | 0.89 |
| *Hypochaeris radicata* | 6 | farm propagation | 14.51 | 56.71 | 0.36 ± 0.10 | 0.64 | 0.69 |
| *Hypochaeris radicata* | 7 | farm propagation | −2.66 | 56.70 | 0.95 ± 0.16 | 0.83 | 0.87 |
| *Lathyrus nissolia* | 1 | greenhouse | 10.76 | 49.02 | -0.03 ± 0.20 | 0.49 | 0.63 |
| *Linaria vulgaris* | 1 | farm propagation | 6.21 | 52.96 | 1.05 ± 0.12 | 0.85 | 0.82 |
| *Linaria vulgaris* | 2 | farm propagation | 11.70 | 48.37 | 0.84 ± 0.15 | 0.80 | 0.81 |
| *Linaria vulgaris* | 3 | farm propagation | 14.48 | 51.87 | 0.21 ± 0.15 | 0.58 | 0.66 |
| *Linaria vulgaris* | 4 | farm propagation | 9.61 | 49.26 | 0.18 ± 0.13 | 0.57 | 0.64 |
| *Linaria vulgaris* | 5 | farm propagation | −2.66 | 56.70 | 1.22 ± 0.15 | 0.89 | 0.91 |
| *Lotus corniculatus* | 1 | farm propagation | 12.61 | 53.87 | 0.46 ± 0.12 | 0.68 | 0.73 |
| *Lotus corniculatus* | 2 | farm propagation | 6.21 | 52.96 | 0.69 ± 0.16 | 0.75 | 0.80 |
| *Lotus corniculatus* | 3 | farm propagation | 11.70 | 48.37 | 0.84 ± 0.11 | 0.80 | 0.88 |
| *Lotus corniculatus* | 4 | farm propagation | 14.48 | 51.87 | -0.01 ± 0.10 | 0.50 | 0.54 |
| *Lotus corniculatus* | 5 | farm propagation | 9.61 | 49.26 | 0.60 ± 0.11 | 0.73 | 0.72 |
| *Lotus corniculatus* | 6 | farm propagation | 9.61 | 49.26 | 0.65 ± 0.17 | 0.74 | 0.83 |
| *Lotus corniculatus* | 7 | farm propagation | 9.27 | 48.27 | 0.53 ± 0.14 | 0.70 | 0.77 |
| *Lotus pedunculatus* | 1 | farm propagation | 8.58 | 49.84 | 1.12 ± 0.07 | 0.87 | 0.91 |
| *Lychnis flos-cuculi* | 1 | farm propagation | 8.58 | 49.84 | 0.31 ± 0.13 | 0.62 | 0.72 |
| *Lychnis flos-cuculi* | 2 | farm propagation | 6.21 | 52.96 | 0.25 ± 0.07 | 0.60 | 0.61 |
| *Lychnis flos-cuculi* | 3 | farm propagation | 13.22 | 46.04 | 1.65 ± 0.12 | 0.95 | 0.96 |
| *Lychnis flos-cuculi* | 4 | farm propagation | 11.70 | 48.37 | 1.45 ± 0.12 | 0.93 | 1.00 |
| *Lychnis flos-cuculi* | 5 | farm propagation | 14.48 | 51.87 | 0.62 ± 0.10 | 0.73 | 0.78 |
| *Lychnis flos-cuculi* | 6 | farm propagation | 9.14 | 50.55 | 0.01 ± 0.24 | 0.50 | 0.60 |
| *Lychnis flos-cuculi* | 7 | farm propagation | 14.51 | 56.71 | 0.86 ± 0.22 | 0.80 | 0.92 |
| *Lychnis flos-cuculi* | 8 | farm propagation | −2.66 | 56.70 | 1.88 ± 0.22 | 0.97 | 1.00 |
| *Medicago lupulina* | 1 | farm propagation | 8.58 | 49.84 | 1.53 ± 0.15 | 0.94 | 0.92 |
| *Medicago lupulina* | 2 | farm propagation | 9.61 | 49.26 | 0.54 ± 0.15 | 0.71 | 0.81 |
| *Medicago lupulina* | 3 | farm propagation | −2.66 | 56.70 | 0.75 ± 0.17 | 0.77 | 0.91 |
| *Plantago lanceolata* | 1 | farm propagation | 8.58 | 49.84 | 1.43 ± 0.13 | 0.92 | 0.97 |
| *Plantago lanceolata* | 2 | farm propagation | 9.18 | 53.28 | 0.90 ± 0.17 | 0.82 | 0.81 |
| *Plantago lanceolata* | 3 | farm propagation | 12.61 | 53.87 | -0.08 ± 0.12 | 0.47 | 0.59 |
| *Plantago lanceolata* | 4 | farm propagation | 6.21 | 52.96 | 1.29 ± 0.12 | 0.90 | 0.96 |
| *Plantago lanceolata* | 5 | farm propagation | 13.22 | 46.04 | 0.56 ± 0.11 | 0.71 | 0.86 |
| *Plantago lanceolata* | 6 | farm propagation | 11.70 | 48.37 | 0.98 ± 0.16 | 0.84 | 0.83 |
| *Plantago lanceolata* | 7 | farm propagation | 14.48 | 51.87 | 1.33 ± 0.19 | 0.91 | 0.98 |
| *Plantago lanceolata* | 8 | farm propagation | −9.14 | 39.40 | 0.85 ± 0.09 | 0.80 | 0.76 |
| *Plantago lanceolata* | 9 | farm propagation | 9.14 | 50.55 | 1.40 ± 0.14 | 0.92 | 0.97 |
| *Plantago lanceolata* | 10 | farm propagation | 14.51 | 56.71 | 1.70 ± 0.19 | 0.96 | 0.98 |
| *Plantago lanceolata* | 11 | farm propagation | −2.66 | 56.70 | 1.51 ± 0.17 | 0.93 | 0.98 |
| *Plantago media* | 1 | farm propagation | 8.58 | 49.84 | 0.16 ± 0.10 | 0.56 | 0.53 |
| *Plantago media* | 2 | farm propagation | 6.21 | 52.96 | 0.88 ± 0.13 | 0.81 | 0.94 |
| *Plantago media* | 3 | farm propagation | 11.70 | 48.37 | 1.24 ± 0.11 | 0.89 | 0.94 |
| *Plantago media* | 4 | farm propagation | 14.48 | 51.87 | 0.21 ± 0.11 | 0.58 | 0.67 |
| *Plantago media* | 5 | farm propagation | 14.51 | 56.71 | 1.36 ± 0.15 | 0.91 | 0.92 |
| *Poa annua* | 1 | farm propagation | 8.58 | 49.84 | 1.93 ± 0.17 | 0.97 | 0.96 |
| *Poa annua* | 2 | farm propagation | 9.18 | 53.28 | 1.89 ± 0.22 | 0.97 | 1.00 |
| *Prunella vulgaris* | 1 | farm propagation | 8.58 | 49.84 | 1.57 ± 0.24 | 0.94 | 0.98 |
| *Prunella vulgaris* | 2 | farm propagation | 6.21 | 52.96 | 0.64 ± 0.12 | 0.74 | 0.84 |
| *Prunella vulgaris* | 3 | farm propagation | 13.22 | 46.04 | 1.21 ± 0.23 | 0.89 | 0.94 |
| *Prunella vulgaris* | 4 | farm propagation | 11.70 | 48.37 | 1.29 ± 0.22 | 0.90 | 0.91 |
| *Prunella vulgaris* | 5 | farm propagation | 14.48 | 51.87 | 1.63 ± 0.21 | 0.95 | 0.89 |
| *Prunella vulgaris* | 6 | farm propagation | −9.14 | 39.40 | 0.59 ± 0.14 | 0.72 | 0.68 |
| *Prunella vulgaris* | 7 | farm propagation | 9.14 | 50.55 | 1.57 ± 0.19 | 0.94 | 0.91 |
| *Prunella vulgaris* | 8 | farm propagation | 14.51 | 56.71 | 1.52 ± 0.15 | 0.94 | 0.88 |
| *Prunella vulgaris* | 9 | farm propagation | −2.66 | 56.70 | 1.09 ± 0.25 | 0.86 | 0.90 |
| *Senecio vulgaris* | 1 | greenhouse | 6.54 | 51.16 | 1.15 ± 0.09 | 0.88 | 0.90 |
| *Senecio vulgaris* | 2 | greenhouse | 8.81 | 50.81 | 1.60 ± 0.26 | 0.95 | 1.00 |
| *Silene vulgaris* | 1 | farm propagation | 8.58 | 49.84 | 1.45 ± 0.12 | 0.93 | 0.96 |
| *Silene vulgaris* | 2 | farm propagation | 6.21 | 52.96 | 2.07 ± 0.13 | 0.98 | 0.99 |
| *Silene vulgaris* | 3 | farm propagation | 13.22 | 46.04 | 2.09 ± 0.13 | 0.98 | 0.99 |
| *Silene vulgaris* | 4 | farm propagation | 11.70 | 48.37 | 1.89 ± 0.12 | 0.97 | 0.97 |
| *Silene vulgaris* | 5 | farm propagation | 14.48 | 51.87 | 1.23 ± 0.18 | 0.89 | 0.88 |
| *Silene vulgaris* | 6 | farm propagation | 9.14 | 50.55 | 0.57 ± 0.12 | 0.72 | 0.68 |
| *Silene vulgaris* | 7 | farm propagation | 14.51 | 56.71 | 1.62 ± 0.17 | 0.95 | 0.99 |
| *Silene vulgaris* | 8 | farm propagation | −2.66 | 56.70 | 2.29 ± 0.22 | 0.99 | 0.99 |
| *Silene vulgaris* | 9 | farm propagation | 12.40 | 49.28 | 1.38 ± 0.18 | 0.92 | 0.96 |
| *Stellaria media* | 1 | farm propagation | 8.58 | 49.84 | 0.14 ± 0.19 | 0.56 | 0.74 |
| *Stellaria media* | 2 | greenhouse | 8.81 | 50.81 | 0.42 ± 0.16 | 0.66 | 0.71 |
| *Thlaspi arvense* | 1 | farm propagation | 6.21 | 52.96 | 0.95 ± 0.22 | 0.83 | 0.88 |
| *Trifolium aureum* | 1 | farm propagation | 12.61 | 53.87 | 0.46 ± 0.18 | 0.68 | 0.75 |
| *Trifolium aureum* | 2 | farm propagation | 11.00 | 49.72 | 0.98 ± 0.13 | 0.84 | 0.80 |
| *Trifolium campestre* | 1 | farm propagation | 8.58 | 49.84 | 1.65 ± 0.18 | 0.95 | 0.97 |
| *Trifolium campestre* | 2 | farm propagation | 11.70 | 48.37 | 1.18 ± 0.20 | 0.88 | 0.92 |
| *Trifolium campestre* | 3 | farm propagation | 9.18 | 53.28 | 1.10 ± 0.13 | 0.87 | 0.89 |
| *Veronica chamaedrys* | 1 | farm propagation | 8.58 | 49.84 | 0.84 ± 0.14 | 0.80 | 0.83 |
| *Veronica chamaedrys* | 2 | farm propagation | 6.21 | 52.96 | 1.62 ± 0.18 | 0.95 | 0.93 |
| *Veronica chamaedrys* | 3 | farm propagation | 11.70 | 48.37 | 1.03 ± 0.14 | 0.85 | 0.90 |
| *Veronica chamaedrys* | 4 | farm propagation | 14.48 | 51.87 | 0.36 ± 0.20 | 0.64 | 0.87 |
| *Veronica chamaedrys* | 5 | farm propagation | −2.66 | 56.70 | 0.92 ± 0.23 | 0.82 | 0.81 |
| *Veronica chamaedrys* | 6 | farm propagation | 12.40 | 49.28 | -0.06 ± 0.12 | 0.47 | 0.60 |
| *Veronica chamaedrys* | 7 | farm propagation | 7.85 | 49.37 | 0.16 ± 0.21 | 0.57 | 0.82 |

**Figure** **S1**. Relationship between the number of seed lots per species and the probability of detecting significant differences among seed lots within species. The line shows a logistic regression with the 95% confidence interval.


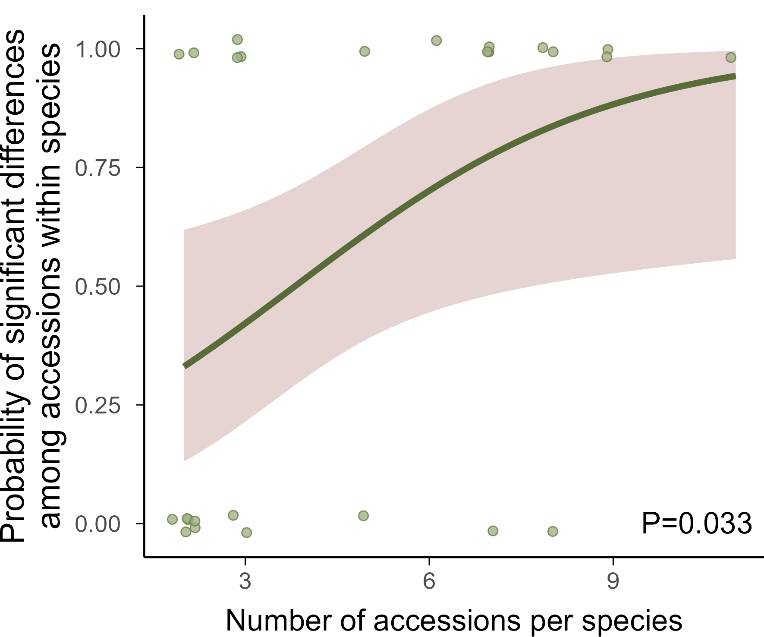

Supplement: Supplementary file 1 — Appendix S1. Supporting tables and figures. Figure S1. Relationship between the number of seed lots per species and the probability of detecting significant differences among seed lots within species. The line shows a logistic regression with the 95% confidence interval. Table S1. Species‐specific seed survival models. Bold type indicates a significant effect of the parameter. A significant interaction between the seed lot identity and the aging duration means significantly different seed longevities between seed lots within species. Table S2. Seed lot information for each species and seed‐lot‐specific seed survival metrics. K i is given on the probit scale and was also back‐transformed on the percentage scale. [file AJB2-113-e70202-s001.docx]
